# Developmental Language Disorder and Autism: Commonalities and Differences on Language

**DOI:** 10.3390/brainsci11050589

**Published:** 2021-04-30

**Authors:** Natasa Georgiou, George Spanoudis

**Affiliations:** Department of Psychology, University of Cyprus, P.O. Box 20537, Nicosia 1678, Cyprus; natasa_georgiou@hotmail.com

**Keywords:** developmental language disorder (DLD), autism spectrum disorder (ASD), comorbidity, autism spectrum disorder with language impairment (ASD-LI), autism spectrum disorder without language impairment (ALN), language development

## Abstract

Language and communication deficits characterize both autism spectrum disorder and developmental language disorder, and the possibility of there being a common profile of these is a matter of tireless debate in the research community. This experimental study addresses the relation of these two developmental conditions in the critical topic of language. A total of 103 children (79 males, 24 females) participated in the present study. Specifically, the study’s sample consisted of 40 children with autism, 28 children with developmental language disorder, and 35 typically developing children between 6 and 12 years old. All children completed language and cognitive measures. The results showed that there is a subgroup inside the autism group of children who demonstrate language difficulties similar to children with developmental language disorder. Specifically, two different subgroups were derived from the autism group; those with language impairment and those without. Both autism and language-impaired groups scored lower than typically developing children on all language measures indicating a common pathology in language ability. The results of this study shed light on the relation between the two disorders, supporting the assumption of a subgroup with language impairment inside the autism spectrum disorder population. The common picture presented by the two developmental conditions highlights the need for further research in the field.

## 1. Theoretical Premises

A growing body of research focuses on the investigation of language and communication deficits observed in children with developmental disorders, such as Developmental Language Disorder (DLD) and Autism Spectrum Disorder (ASD) (e.g., [1,2]). Individuals diagnosed with DLD are found to have difficulties in areas related to the structure of a language, such as phonology, morphology, syntax, and semantics. It has been suggested that individuals diagnosed with ASD have difficulties in another category associated with language, namely pragmatics. Pragmatics resides at the top of the hierarchy of the building blocks of language and is defined as one’s ability to use the language appropriately in each context [3]. Deficits in this last component of language led some researchers to the conclusion that structural language deficits may also be present in ASD [4]. Nowadays, the research community has raised its interest in whether ASD and DLD disorders overlap, and examines the possibility that these two disorders may consist of different manifestations of the same underlying cause.

### 1.1. Developmental Language Disorder

Developmental Language Disorder (DLD) is not a term included in the Diagnostic and Statistical Manual of Mental Disorders (DSM-5), however, it is widely accepted amongst both the scientific community and clinicians. Instead, the DSM-5 includes the term “Language Disorder” (within neurodevelopmental disorders) to define persistent difficulties in the acquisition and use of language, because of impairments presented in comprehension or production [5]. Until recently, it was widely referred to as “Specific Language Impairment (SLI)”. Nevertheless, in the recent past the term proposed by Bishop and colleagues [6], namely “Developmental Language Disorder” (DLD; CATALISE-2 consortium; Criteria and Terminology Applied to Language Impairments: Synthesizing the Evidence), has been widely accepted and used extensively. In the present study, we will refer to this disorder using this term (Developmental Language Disorder; DLD). The symptomatology of DLD includes difficulties that create obstacles to communication or learning in everyday life, and those language problems are unlikely to resolve or will have not been resolved by five years of age and are not associated with a known biomedical condition such as brain injury, neurodegenerative conditions, genetic conditions or chromosome disorders such as Down syndrome, hearing loss, autism spectrum disorder or intellectual disability [6].

DLD is a heterogeneous disorder; symptoms can be either expressive (e.g., syntax, vocabulary, phonology, and motor skills), receptive (i.e., comprehensive skills), or a combination of the two [7]. Recently, more empirical evidence supports that children with DLD are also deficient in pragmatics, a non-core feature of language [8]. It has also been suggested that a subtype of DLD exists and presents reduced pragmatic abilities [9].

### 1.2. Autism Spectrum Disorder

Autism is a disorder characterized primarily by social deficits, as two of the three main diagnostic features, namely impaired communication and reciprocal social interaction, exist within the social domain. According to the DSM-5, a diagnosis of ASD has to include deficits in (1) social communication, and social interaction which occur across multiple contexts and (2) restricted repetitive behaviors, interests, and activities [5].

Although structural language impairment as part of a communication deficit, is not a necessity for an ASD diagnosis (according to DSM-5), it is strongly associated with ASD [4]. Specifically, it has been stated that approximately 63% of all children diagnosed with ASD have language impairment [10]. It has also been found that over half of the individuals with ASD have additional deficits in other levels of the structure of a language, such as in phonology, grammar, and semantics [11]. A delay in language acquisition and a lack of the ability to use language appropriately in social contexts (lack of pragmatic abilities) are the core features of ASD. Pragmatic abilities are a hallmark feature of the disorder, regardless of an individual’s level of functioning [2,12].

ASD is a highly heterogeneous disorder, with a wide range of abilities in language and cognition throughout the spectrum. Understanding the nature of these deficits is complicated by the great heterogeneity of language and cognitive abilities within this population [2]. For instance, although many ASD individuals present expressive and receptive language impairments, there is also an important number of ASD individuals who do not display any language deficits (ALN subgroup) [13]. This variability complicates the effort to understand the nature of the cognitive and language deficits of the disorder.

**Figure 1:**
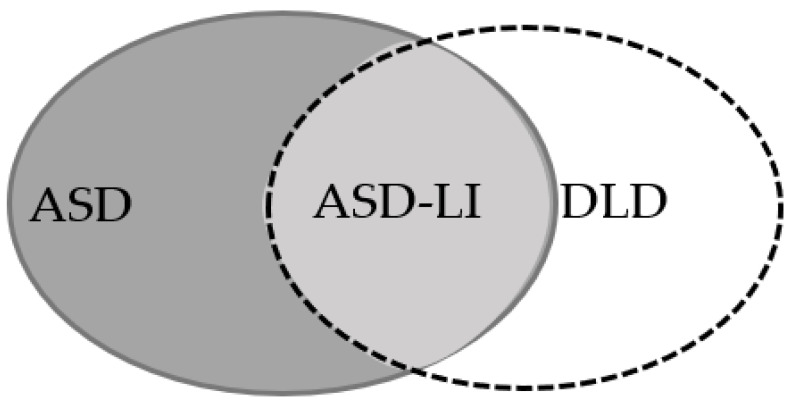
A depiction of the proposed intermediate category ASD-LI, sharing features from both ASD and DLD.

Language impairment is the primary symptom of DLD condition. As previously mentioned, studies are supporting the existence of language deficits in ASD individuals [14,15,16]. This finding triggered the scientific community to further investigate the relation of the two developmental conditions in the domain of language. Research evidence so far has provided some interesting findings pointing at commonalities in the language domain [15,16]. Data from the use of language tasks along with data from cognition neuroimaging and genetics in ASD children provide evidence for the existence of an ASD-LI (ASD plus Language Impairment) subgroup within the ASD group [14,15,16]. Figure 1 represents the ASD-LI “subgroup” of shared symptoms between the two disorders.

**Figure 2:**
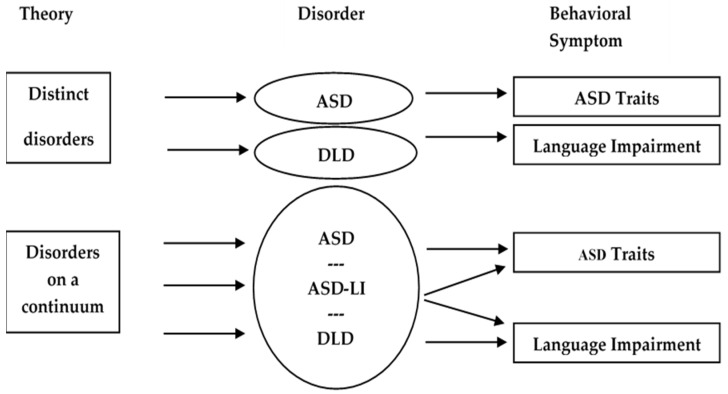
Models of the relationship between DLD and ASD. They depict different theories explaining the relation between ASD and DLD.

In this figure, an overlap is identified between the two disorders. This overlap, this common area between the two disorders has been documented by different studies despite the widely accepted view of them being characterized by different symptoms [14,15,16]. A possible link between ASD and DLD has been stated as a matter of investigation for additional research. Studies to date had allowed researchers to draw different conclusions based on controversial findings. Currently, there are two key assumptions within the research community addressing the relation of ASD and DLD. The two different perspectives are presented in Figure 2.

In particular, one part of the research community argues that the two disorders are distinct and attribute any common ground to superficial similarities; (e.g., [2,13,17,18]) while the other part of the community argues that the two disorders consist of different manifestations of the same pathology and lie on the same continuum [19]. This second assumption has received important support from researchers supporting that ASD and DLD disorders are comorbid (e.g., [15,16]).

The debate regarding whether there is a common etiological phenotype between the two disorders is still ongoing. Research so far cannot provide conclusions on the similarities and differences between the two disorders. The need for more research in the area is underlined, to provide evidence either to support or dismiss the assumption of a common etiology behind DLD and ASD.

### 1.3. A Potential Overlap between ASD and DLD: Language Development in DLD 

Language problems are common in children, with DLD prevalence estimated to be 7.5% [20]. Children with DLD present impairments in language acquisition and use, despite having normal nonverbal intelligence and in the absence of other significant developmental/hearing deficits, autism, and severe neurological impairment [21]. Language abilities of individuals with DLD are assessed through multiple measures of both expressive and receptive language. Children with DLD demonstrate deficits in verbal working memory (e.g., [22,23]) and on nonverbal as well [23,24]. 

Research so far, in an attempt to identify the specific causes of DLD, has pointed out some clinical markers, related to DLD deficits [25]. Specific errors of tense marking (i.e., omission of the past tense marker, 3rd person present tense marker) [26], poor sentence repetition [27], and poor non-word repetition are all considered as discriminative markers of DLD children from typically developing (TD) children [28]. 

Specifically, tense marking ability has been documented as a DLD indicator by the early studies of Rice et al. [29] and Marchman et al. [30]. It has been identified as a key feature of children with DLD, examined by an elicitation task, and a task of the use of third person singular [31]. Children with DLD were also found to demonstrate poor performance on syntactic tense throughout the primary school years [30]. Furthermore, sentence repetition is a good indicator of DLD, for children speaking English [27] or dialects of English [32], also Cantonese [33], French [34,35], and Greek Cypriot [36].

For example, in the first study conducted in Cyprus, measuring and validating Sentence Repetition Task (SRT) in Greek-Cypriot children, results showed significant differences, as the group of children with DLD scored poorer than the TD group of children participated in the study [36]. The results from the English speaking population are reported by Conti-Ramsden et al. [27], who investigated different potential clinical markers of DLD and found that sentence repetition (also third person singular task, tense marking, and non-word repetition) could be the strongest clinical marker for the identification of DLD. A repetition task considers contributing to the identification of children’s weaknesses and strengths in the language domain [37].

Another important clinical marker in the field is non-word repetition. A majority of research has pointed out a strong connection between consistently poor performance on non-word repetition tasks and DLD (see [38]). In these tasks, listeners are first asked to hear a made-up word modeled after their native language, (e.g., “vonk” in English, or “Βηκα” in Greek), and then, repeat it back immediately. The non-word repetition task is considering an excellent clinical marker to distinguish DLD individuals from typically developing individuals [1,28].

Especially, non-word repetition tasks received a great deal of attention in investigations of language impairments. Indeed, their connection to language impairments occurs reliably across the literature [39,40]. Non-word repetition tasks have received significant attention in studies of language impairments because of their ability to comprehend the underlying deficits of children with DLD and acts as a possible identifier of these children. A meta-analysis provided evidence from different studies indicating that children with DLD displayed difficulty repeating not only long non-words but even short nonwords [40].

Tasks such as Non-Word Repetition (NWR) and Sentence Repetition (SNT), have received great research interest as psycholinguistic markers of DLD [27,41,42]. NWR and SNT measures are used as phonological short-term memory indicators, and putative markers for DLD [41]. NWR can discriminate DLD from other childhood communication disorders [42] and exceeds other language measures such as past tense marking in its specificity and sensitivity to detect language-impaired individuals [27].

In addition to the above, it is also important to make a brief reference to the field of pragmatic language and its relation to DLD. Pragmatic language is strongly associated with deficits presented in ASD individuals, whereas, its relation to individuals with DLD ability, is still not determined. On the one hand, studies are supporting additional pragmatic impairments in children with DLD [3,43,44] while on the other, evidence of intact pragmatic ability, is provided [45,46], highlighting the need for more research in the field. 

DLD has been extensively studied in several different languages, and interesting results emerged from studies conducted in the Greek language. Studies in Greek-speaking children with DLD revealed important results especially in relation to specific domains of linguistic or cognitive difficulties.

The main findings indicate difficulties of children with DLD in their ability to comprehend thematic roles, namely, grammatical subject–object relations in complex main-relative clauses, by utilizing pro- and post-verb clitics about their peers [47], and in the production and understanding of pronouns [48,49]. In addition, longitudinal studies inform for persistent impairments in specific aspects of language, such as persistent problems with specific grammatical operations 5 years later [50,51]. 

Interestingly, controversial results are reported by studies investigating clitic production in Greek-speaking children with DLD. Studies are reporting that children with DLD omit direct-object clitics [52,53] while others report no differences between children with DLD and TD peers in similar tasks [54,55,56]. Narratives have also been tested in Greek-speaking children and found to be effective in differentiating DLD children from TD [57]. The authors noted that narratives can indeed be a useful tool to identify and assess language-impaired children in the Greek language.

Additional findings support that children with DLD have difficulties in understanding the semantic and pragmatic functions of mental state verbs and working memory [58]. Mainly, research on the Greek language specifies difficulties that children with DLD perform in semantics, morphosyntax, and pragmatics and not necessarily in phonology (see also [59]).

The majority of research in the Greek language focuses on specific areas of linguistic ability. Nevertheless, more research is needed related to the linguistic abilities of DLD children on all linguistic domains, especially in comparison to the abilities presented by ASD children, to effectively locate the similarities and differences of the two disorders and shed light on the predominate “comorbidity” question.

### 1.4. Language Ability in ASD

Language ability in autism was described from early on as impaired, especially concerning the social use of language and to the quality of the spoken language (e.g., [60,61]). However, not much investigation conducted in the following years related to the language deficits in autism, possibly because early studies did not reveal any significant differences in linguistic domains of phonology, syntax, and morphology, between children with ASD and TD children, matched either on age, IQ or language (e.g., [62,63,64,65]). 

Research so far provided evidence of higher-verbal children with ASD, who score similar to TD children, based on a range of structural language measures [66]. The ability in phonology, semantics, morphology, and syntax of a subgroup of ASD individuals is furthermore supported by different studies [67,68]. It has been found that there are individuals with ASD who present good or even elevated structural language abilities on formal testing, by using sophisticated vocabulary and sentence structure [69,70]. 

The great heterogeneity observed within the ASD population is also apparent in the extent of structural language difficulties that co-occur with ASD [4,66,71]. It is argued that a percentage of 25% and 30% of children with ASD are minimally verbal or nonverbal, based on studies ascertained via population-based or clinical samples [72,73]. The incidences presented may vary according to individuals’ age, different study criteria of language ability definitions, and by the way the sample was ascertained [74]. Nevertheless, impaired language development is often the first irregular symptom to be identified by parents of children who are later on diagnosed with ASD [75,76]. It has also been found that over half of the individuals with ASD have additional deficits in different levels of the structure of a language, such as in phonology, grammar, and semantics [11]. In addition, the progress of lower verbal children with ASD, found to be slower, and also presenting flatter language development [66]. 

As it concerns the communication domain, diagnostic features refer to delays and impairments in language acquisition, and qualitative deficits in discourse and other pragmatic aspects of language as well. A great emphasis in the research community about language in autism has focused its interest on the pragmatic impairments, which are treated as universal among ASD individuals and are specific to this disorder [4,77]. The pragmatic impairment, is a hallmark feature of the disorder, regardless of an individual’s level of functioning [2,12].

In the Greek language, only limited studies included children with ASD. In a study investigating the narrative skills of 30 Greek-speaking children with ASD (ALN and ASD-LI) and a TD group, their narrative ability was measured in terms of both microstructural and macrostructural properties. Microstructural properties are referring to lexical and syntactic measures of complexity and macrostructure to the diversity in the use of internal state terms (ISTs) and the complexity of the story’s structure. The results showed that increased language ability and syntactic complexity are positively connected to ASD children’s narrative performance. However, both ALN and ASD-LI groups of children with ASD scored lower than the TD controls in measures of the production of Theory of Mind-unrelated ISTs, modifier clauses, and story structure complexity [78].

In an additional study in the Greek language with high-functioning ASD children, results were indicative of difficulties in the ASD group at the interface of syntax with pragmatics and prosody. Specifically, they produced significant deficits in their ability to distinguish a discourse prominent element and to consider intonation relevant for a particular interpretation that excludes clitics, in relation to their TD peers [79]. 

The difficulties of ASD-LI children in the Greek language are also supported by a study with Greek-Cypriot children investigating comprehension and production of two-constituent compound words. The results of the study indicate that despite the ASD-LI children’s ability to recognize compound constituents they demonstrated impairments to understand the compound meaning. In addition, they produced semantically inappropriate responses when attempted to explain the meaning of the compounds, indicating limitations in their conceptual-intentional system [80]. 

To sum up, ASD is a highly heterogeneous disorder, with impaired cognitive-language abilities throughout the spectrum. Understanding the nature of these language deficits is complicated by the great heterogeneity of language and cognitive abilities within this population [2]. For instance, although many ASD individuals present expressive and receptive language impairments, there is, simultaneously, an important number of ASD individuals who do not display any language deficit [13]. This variability complicates the effort to understand the nature of the cognitive and language deficits of the disorder. More research is necessary to clarify the nature of the language impairments in (subgroups of) children with ASD, and how it may be related to that presented in other developmental conditions of language pathology, such as DLD.

### 1.5. ASD and DLD Children’s Relation on Language Ability

Many studies have examined any potential phenotypic overlap between autism and DLD, especially in the language domain. Numerous studies have found that there are many similarities in the language abilities of children with autism and children with DLD and argue that the reason for these similarities is a shared etiology [14,15].

#### 1.5.1. Similarities

Impaired language development is apparent in DLD and—at least—in subgroups of ASD individuals. The relationship though, of these two developmental conditions with language deficits, is still undetermined. In the last twenty years, several assumptions have been made suggesting that a clear diagnostic border separating the two disorders may not emerge as clear as expected [15,16,81]. This hypothesis is supported by studies concluding that there are common features between the two disorders [15,16]. Furthermore, Lindgren and colleagues in their 2009 study discovered no statistically compelling differences between the two groups in a variety of language domains. Those discoveries of language similarities, gave voice to the researchers to contemplate that there is a possible shared etiology at the background of the two disorders [82].

In addition, Lloyd and colleagues [83] administered a standardized language test to three groups of children, ASD, DLD, and a shared group with common symptoms from both disorders. The results provided mixed results. Despite that, the composite scores of the “shared” and ASD groups had the most similarities, and the DLD group had the lower expressive language scores, all three groups had the least difficulty with “Listening to Paragraphs” (which involves abstracting and remembering information from two short texts). It also showed that all three groups had difficulty with “Recalling Sentences” (SRT; which involves repeating sentences of increasing length and complexity). Comparable performance in expressive language between the ASD and DLD groups measured by the task of Repeating Sentences has also been stated by Manolitsi and Botting study [84]. In addition, others demonstrated similar phonology [85] and vocabulary delays [85,86]. 

The assumption of a common phenotypical path linking the two developmental conditions is furthermore strongly supported by McGregor and colleague’s findings [86]. In their study, they compared an ASD group of children, who were free of syntactic deficits and had age-appropriate word knowledge, a group of children with ASD plus concomitant syntactic language impairments (ASD-LI), a DLD group of children, an age-matched group (AM), and a syntax-matched group (SM), both consisted by unaffected peers. Their results showed that ASD-LI children, performed similarly to the DLD group, and both showed sparse lexicons with partial word knowledge and immature knowledge of word—relationships. The authors state for a behavioral overlap related to the syntax–lexicon interface and point to the possibility of a commonality in the ASD-LI and DLD phenotypes.

The grammar ability of the two groups has also been studied by Tuller and colleagues [87], who stated that both ASD and DLD participants make mistakes with clitic pronouns and complex sentences. They also reported that the ASD-LI group (and the children with DLD) were particularly sensitive to consonants in syllable final position, notably liquids and obstruents in internal coda position when examined on a standardized word repetition task. ASD-LI and DLD groups were identified to develop the same strategies in an attempt to avoid syllabic complexity, such as the omission of a segment. Riches and colleagues [18] also reported that syllabic structure is problematic for adolescents with ASD plus language impairment.

More recent data also highlight the possible overlap between the two conditions. Specifically, the CELF-4 standardized test was administered to assess ASD, DLD, and TD children’s language skills. The results showed no significant differences in language between the DLD and ASD groups, suggesting an overlap in the linguistic profiles of children with DLD and children with ASD [88].

Recent evidence supports the existence of common ground between the two conditions in relation to the field of semantics. Gladfelter and Barron [89] explored whether global–local processing differences influence the type of semantic features children with ASD, DLD, and typically developing peers learn to produce when learning new words. The results showed that the children from the clinical groups produced more global, in relation to local, semantic features in their definitions than the TD group. This finding further supports a common feature in semantic language and may reflect common deficits in depth of word knowledge. 

In addition, a study by Modyanova and colleagues [90] investigating the performance of ALN and ASD-LI children in their ability in tense marking and morphosyntax revealed a clear distinction in the morphosyntactic abilities of the two subgroups. ASD-LI scored significantly worse with grammatical ability. The ASD-LI profile was closer to the one usually performed by the DLD children, and perhaps even more impaired. A study comparing ASD and DLD groups on tasks of working memory and morphosyntactic processing showed that children in both groups were more accurate and faster at detecting errors occurring late, rather than early, in the sentence, indicating no evidence of different patterns of performance for the DLD and ASD groups [91].

There is also research evidence supporting the existence of a common ground related to the field of pragmatics. In a recent study, where ASD-high function (HFA), DLD and TD groups of children participated, the groups were experimentally tested on various components of grammar, pragmatics, and nonverbal cognition. The results showed similar performance of DLD and HFA in pragmatics, lower than their TD peers. However, the DLD group performed significantly lower than the TD children on grammar and several cognition tests, while the HFA group did not [92]. Thus, although a similar profile is identified on pragmatics, differences are also noted by this study. 

Additionally, Norbury and colleagues [93] also investigated pragmatic language and the narratives of ASD, DLD and TD children and revealed no significant differences between children with ASD and DLD, who produced similarly simple narratives that lacked semantic richness and omitted important story elements, when compared to TD peers. Importantly, pragmatic errors were common across groups.

As it concerns the longitudinal outcomes of the proposed overlap, data from adolescents come to add to the “commonality” hypothesis. Specifically, Conti-Ramsden and colleagues [94], in their study, found that 3.9% of teenagers previously diagnosed with DLD, showed sufficient characteristics of autistic behavior and met the criteria for an autism diagnosis. Leyfer and colleagues [95] determined that a percentage of 41% of a DLD diagnosed group of children, reached the diagnostic boundaries for ASD in social and communication domains, and also displayed impairments in structural language. In line with the previously mentioned discoveries, data from long-term studies provide evidence that individuals who were previously diagnosed with DLD, as teenagers and adults, express characteristics that are similar to autism [96,97]. Those findings could imply a possible relation between the two disorders, which becomes more apparent with children’s growth, as social expectations grow as well, and their DLD symptoms alone cannot explain their social deficiencies. Then it seems that their symptoms might become more autistic-like.

#### 1.5.2. Differences

Despite the above-mentioned studies arguing in favor of possible comorbidity of the two conditions, another theoretical position states that the similarities in the language and memory performance of children with autism and children with DLD are simply superficial, and do not suggest a common etiology [98]. 

The evidence for this position gains support from studies in which children with DLD and children with autism demonstrate different patterns of deficits between the two conditions (e.g., [2,13]). For instance, Demouy and colleagues [85] examined receptive and expressive language in children with ASD, DLD, and Pervasive Developmental Disorder Not Other Specified (PDD-NOS) and reported that skills in ASD and DLD rely on different mechanisms, while PDD-NOS showed an intermediate profile sharing some characteristics of both AD and DLD. The results identified significant differences between three groups in syntax, pragmatics, and prosody. DLD demonstrated correlations between chronological age and raw scores in all language tasks, while the two other groups did not. In addition, DLD showed correlation within all raw scores in language tasks, the majority of correlations were also found in PDD-NOS but not in the ASD group. The authors suggest that language skills in ASD and DLD are not comorbid, however, they argue about the existence of some common symptoms, as they consider PDD-NOS as an intermediate profile sharing some characteristics of both ASD and DLD. 

In another study with adolescents with DLD and ASD-LI, the findings showed the second groups’ participants to outperform DLDs’ in lexical tasks involving word associations and structures [83]. In an additional study with adolescents, Loucas and colleagues [99] investigated three groups of ASD-LI, DLD, and TD adolescents, in tasks of spoken word recognition with frequency (low/high) differences and several phonological onset neighbors. They found that adolescents with ASD-LI needed more speech to identify low-frequency words with opponents of low density, than the other two groups, which showed a similar performance, pointing to differences between clinical conditions. 

Furthermore, data derived from the field of non-word and sentence repetition tasks provide important evidence related to the nature of the commonalities observed. Some studies have been found different patterns of errors related to ASD and DLD children, on non-word and tasks which require repetition of sentences [13,18]. Specifically, Whitehouse and colleagues [13] claim that even if some common deficits do exist in both disorders, there are different underlying causes behind those. They have disclosed different kinds of non-word repetition mistakes between the two groups, concluding that there is no evidence supporting that those two disorders may have a common behavioral or etiological result. However, this result should be viewed with caution, because the number of participants in the ASD group who performed poorly enough on the non-word repetition measure and was included in the analysis was small (*n* = 8). An additional study [99], despite concluding on the same findings as Whitehouse and colleagues [13], did not find any compelling differences between the two groups concerning their overall error rates, and their percentage of phonemic mistakes that specifically affected the syllable structure of the stimuli, on word-repetition performance. Though, again, the results should be seen with discretion because ASD-LI children were not matched on their chronological or verbal mental age (VMA) to DLD children [99].

The assumption that ASD participants demonstrate higher performance than DLD participants in nonword tasks is furthermore supported by other scientists [17,18]. For instance, Durrleman and Delage [17] reported that both groups, ASD and DLD, performed worse than TD on NWR tasks, of the same chronological age. Group differences reveal the most pronounced impairments relate to the DLD group since the children with ASD performed superior to the group with DLD. These authors also reported similar deficits for third person accusative clitic production and general morphosyntax between the two groups and stated the existence of a subgroup of children with ASD, which showed intact grammar except for 1st person accusative clitic production, where children with DLD showed good performance. 

A more recent study compared the performance of individuals with ASD with individuals with Syntactic-Developmental Language Impairment (SyDLI) and a TD group. their results support that although the two clinical groups show similar performance in syntactic tasks, they produced different error patterns [100]. Creemers and Schaeffer [101] showed another demonstration of the differences between the two groups in a Dutch-speaking population. ASD and DLD participants were compared to a lexical-syntactic task of mass-count distinction, and a pragmatic task (testing the use of definite markers). The results showed that the ASD participants perform better than the DLD participants on the grammatical mass-count task, similar to the TD level. They performed, however, worse than the DLD group when they were asked to provide a definite determiner, a task that requires pragmatic abilities [102,103]. 

In addition, data from pragmatic language and narrative tasks reveal different levels of performance between the ASD and DLD children. For instance, in a study using pragmatic language and narrative tasks, different levels of performance were produced between the ASD and DLD children, and narratives showed that ASD children produced more expressive mistakes during their story-telling compared to DLD children. Furthermore, ASD children were found to produce more receptive but not expressive mistakes compared to DLD children [84]. This finding is also supported by Hudry and colleagues [104] who found the receptive language abilities are more impaired than the expressive language abilities, in a group of pre-schoolers with autism. These findings led researchers to hypothesize that there are distinct etiologies explaining these deficits. In a review of the literature, Williams and colleagues [1] concluded at this second position, arguing that there is not much evidence supporting a shared etiology explaining their language deficits.

Given that the above studies point to a different phenotypical path, researchers are left to hypothesize that distinct etiologies are explaining these deficiencies. Still, there is not enough evidence to explain the origin of the similarities presented by the two developmental conditions. Recently, Taylor and Whitehouse [105] reviewed studies investigating overlaps in the phenotypes of DLD, ASD, and social (pragmatic) communication disorder (SPCD). They concluded that some children with language impairments will fall between the boundaries of conventional diagnostic criteria for ASD and DLD and could meet the criteria for SPCD. The authors argue that it may be the case for DLD, SPCD, and ASD to be related disorders that vary according to the degree of the presented deficits in different domains; namely structural language, pragmatic language, and circumscribed interests (also see [106]). 

This possibility of relatedness as it derives from their similarities enhances the question as to whether DLD and ASD are parts of the same continuum, as Bishop supports [19]. The observed similarities between the two developmental conditions, let Bishop outbid in favor of a shared etiology of the two. She then argues that DLD and ASD constitute points on a continuum of the same disorder instead of separate conditions [19]. 

Considering all the above mentioned, we can conclude that, the commonality picture is blurred and the available research data provide conflicting results. We can conclude that there is important room for research, to deeply comprehend the relation between the two disorders, especially, in the language domain.

### 1.6. The Present Study

#### 1.6.1. Purpose of the Study 

The importance of locating the similarities and differences of DLD and ASD developmental conditions is critical, to clarify the specific symptoms of each disorder and intervene accordingly. Despite the research efforts which have been made so far, the question is still open. Putting the findings together, it seems that there is some evidence supporting the possibility of an overlap between the two conditions in language domain.

Hence, this study aims to further investigate whether the observed similarities between the two disorders are surface manifestations of the same underlying neuropsychological dysfunction, or whether they derive from distinct pathologies. The relation between DLD and ASD will be extensively investigated in relation to the critical domain of language.

#### 1.6.2. Hypotheses

We hypothesize that an ASD-LI subgroup of children will be formed, based on participants’ performance on the Expressive and Receptive Language Evaluation (EREL) [107], a measure which represents the Greek version of the CELF instrument and is suitable to identify children with DLD.

We also hypothesize that ASD (ASD-LI and ASD without Language Impairment; ALN) and DLD children will score lower than typically developed group (TD) on language measures. Specifically, we expect that ASD-LI and DLD groups to demonstrate poorer performance than ALN and TD groups on language tasks, and also the ALN group to score poorer than the TD group.

We hypothesize, especially, that the “Sentence Repetition Task” (SRT or RS; EREL instrument) will differentiate significantly the three clinical groups from the TD children, as it is considering to be an important instrument in the diagnostic process of DLD [2]. SRT can also provide information about the language profile of children and their language strengths and weaknesses [36]. Similarly, the Non-Words Repetition task (NWR) along with the SRT, is expected to accurately locate language impairments, if any, in the experimental groups, as both measures have received great research interest as psycholinguistic markers of DLD [27,41,42]. If there is a linguistic overlap in ASD-LI and DLD, then, in comparison to ALN and TD children, we expect both the ASD-LI and DLD groups to perform significantly worse on NWR and SRT tasks.

We also expect children’s symptoms in each group to reflect on the primary deficits of each disorder. Especially, DLD children are expected to present difficulties primarily on their general communication ability, while ASD children will demonstrate additional impairments in the social interaction ability, based on the primary deficits of each disorder. These abilities will be tested by the Children Communication Checklist Second Edition [108].

## 2. Materials and Methods

### 2.1. Participants’ Recruitment

A total number of 103 children participated in the study (79 males, 24 females). Participants were 40 children (38 males, 2 females) referred by special educators and speech pathologists as ASD children (initial number tested 41 children, one child excluded), 24 children as DLD children (14 males, 10 females), and 39 TD children (27 males, 12 females) were recruited from the general population referred as a control group, aged 6–12 years old. 

ASD and DLD participants were all recruited from private practices in Cyprus, located in different parts of the main cities in Cyprus to represent as much as possible different demographics in our study. The speech pathologists and special educators of children informed their parents if interested in attending the study. Specialists were specifically informed about the child characteristics which fitted this study’s profile. They were, specifically, instructed to request the parents’ availability for participation if their children were already diagnosed with ASD or DLD, or if the child’s characteristics were resembling such a profile. If parents were interested the researcher contacted them providing them with further information about the study. In the ASD referred group, an ASD diagnosis was needed, from a licensed psychologist or psychiatrist, trained in the clinical administration of the Autism Diagnostic Observation Schedule, Second Edition [109]. 

The children in the two clinical groups were recipients of special education services. From the initial DLD group, all of the participants were receiving speech therapy. From the ASD group, the majority of children (*n* = 32) were involved in speech therapy, to address different deficits in language, related to different components of language, such as in structural and pragmatics domains. 

In addition, a comparison group of children from the general population was formed. These children were selected randomly, from children aged 6–12 years, attending public schools in Cyprus. 

For all the participants’, their relatives (mothers, *n* = 103) were also included in the study. Permission from the Cyprus National Bioethics Committee and parental consent for participation in the study were obtained before testing. Children’s verbal agreement was also obtained before testing.

#### 2.1.1. Demographic Characteristics

Participants are all Greek native, from mixed socio-economic backgrounds.

#### 2.1.2. Group Selection

DLD: The children met the inclusion criteria for Developmental Language Disorder described in the Diagnostic and Statistical Manual of Mental Disorder (5th edition) [5]. Specifically, participants in the DLD group were able to participate, if they fitted on DLD diagnostic criteria of the study when administered the EREL measure. Participants who scored 1.25 standard deviation below the mean, were suitable for inclusion in this group. The children’s cognitive ability was also assessed, to reinsure that no participant fell in an intellectual disability score (75 or above), based on their performance on the Wechsler Abbreviated Scale of Intelligence First Edition (WASI) [110]. Two participants had a score of 71, however, given that the IQ score obtained was general and not only their Nonverbal ability, but we also reached the maximum criterion of inclusion in the study.

Our decision to include participants with IQ scores around 75 is supported by several studies in which scores below and including 75 were allowed in language impairment samples [111]. In addition, it remains an unresolved question whether would have been more effective to expand the lower level of IQ score as low as 70 or below, thereby introducing greater variability within the group of children with language impairments. One statement is that there are no interesting language differences between groups defined according to the conventional criterion (Nonverbal IQ of 85 and above) and the expanded criterion (Nonverbal IQ 70 and above) [112]. Importantly, under the application of DLD criteria, children with nonverbal IQ scores below-average are not excluded, unless those scores are so low as to merit the diagnosis of intellectual disability [113]. Nevertheless, in this study, the threshold of 75 score which was set, was assessed for the general IQ ability of the participants, and consequently, we can assume that for this deficient group, their Nonverbal ability will be greater. 

ASD: Children have to meet the inclusion criteria for Autism Spectrum Disorder described in the Diagnostic and Statistical Manual of Mental Disorder [5]. Participants in the ASD group were able to attend the study if they fitted an ASD diagnosis (A formal ASD diagnosis by licensed psychologists or psychiatrists, trained in the clinical use of the instrument ADOS-2). ASD children’s cognitive ability was within the normal range (75 or above), based on the WASI [110]. 

#### 2.1.3. Exclusion Criteria

Exclusion criteria were IQ scores under the average and any other history of psychiatric or medical conditions. Additionally, children with other neurologic impairment, global developmental delay, or significant hearing impairment were excluded from the sample. Children whose parents are unwilling to complete the caregiver’s measures were also excluded from the study. Based on the above criteria, one child was excluded from the research, because it was not able to complete the WASI test.

The following section describes the different instruments and measures administered. All of the administrations were performed by the researcher, who has the qualifications to assess and provide diagnosis, as a licensed school psychologist. The overall examination had a duration of approximately 2.5 h, with a 10 min break in between. The mothers of the participants were simultaneously completing the measure administered to them unless they needed some additional help or clarifications from the researcher. In the second case, after the completion of the child’s participation, the researcher provided the appropriate guidance and help to the mothers to complete their measures. 

### 2.2. Measures

#### 2.2.1. Pre-Test Examination Measure

Wechsler Abbreviated Scale of Intelligence First Edition (WASI) [110]. This scale is used to measure participants’ IQ and to ensure that no child presents intellectual disability among participants. The two-subtest short version will be utilized (vocabulary and matrix reasoning), and it is administered to provide a comprehensive picture of the participants’ general cognitive ability.

#### 2.2.2. Assessment Measures

Expressive and Receptive Language Evaluation [107] (EREL; Greek standardized test similar to Clinical Evaluation of Language Fundamentals, Fourth Edition, CELF-4 [114]. This test is a suitable instrument to assess language ability and it is administered to individuals from 5 to 12 years old. In this study, participants will be assessed with this instrument to detect their linguistic abilities. Individuals need to respond verbally to stimuli presented in pictures. The Core Language score for the respective CELF-4 presents high sensitivity of 0.86 and specificity of 0.96 [114]. The reliability index of the EREL was ranging from a = 0.78 to a= 0.96 on the Core Language score for the age groups represented in our study [107]. The Core Language subtests of the EREL were administered and are referring to: 

(i) Concept and Following Directions, a subtest that examines participants’ ability to (a) interpret spoken directions of increasing length and complexity; (b) follow the stated order of mention of familiar shapes with varying characteristics such as color, size, or location; and (c) identify from among several choices the pictured objects that were mentioned. These abilities reflect short-term and procedural memory capacities.

An example of such a task is: (instructions: “show the sock and then show the bird. Let’s go”).

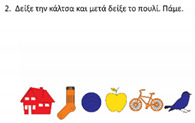



(ii) Word Structure, which aims to evaluate the participants’ ability to (a) apply word structure rules (morphology) to mark inflections, derivations, and comparison; and (b) select and use appropriate pronouns to refer to people, objects, and possessive relationships. 

An example follows where the administer reads aloud the instructions and the participant only see the picture presented below. Instructions are given first for the picture on the left, “Here we have a fox”, and then we show the picture on the right. “Here we have two….” (and we expect the child to say “foxes”).

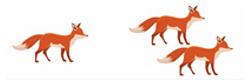



(iii) Formulated Sentences, to investigate the ability to formulate complete, semantically, and grammatically correct, spoken sentences of increasing length and complexity (i.e., simple, compound, and complex sentences), using given words (e.g., car, if, because) and contextual constraints imposed by illustrations. These abilities reflect the capacity to integrate semantic, syntactic, and pragmatic rules and constraints while using working memory.

In the following example, participants are asked to create a sentence by given the word “book” (“βιβλίο” in the Greek language).

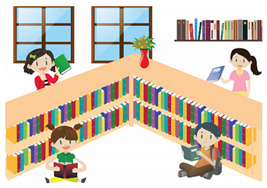



(iv) Recalling Sentences, is a subtest by which the participant is asked to listen to spoken sentences of increasing length and complexity, and repeat the sentences without changing word meaning and content, word structure (morphology), or sentence structure (syntax). Semantic, morphological, and syntactic competence is examined, as it facilitates immediate recall (short-term memory). 

Some examples of sentences to recall are:

“Ζήτησε να έρθει μαζί μας γεμάτος χαρά” (He asked to come with us full of joy).
“H κατάσταση ήταν πραγματικά πολύ άσχημη για όλους” (The situation was very bad for everyone).
“Aποφάσισαν ότι το χωράφι θα πουληθεί και τα λεφτά θα μοιραστούν στα τρία αδέλφια και τις δύο αδελφές” (They decided that the field will be sold and the money will be divided to the three brothers and the two sisters), and

(v) Word Classes, aiming to evaluate the participants’ ability to analyze words for their meaning features, define words by referring to class relationships and shared meanings, and describe meanings that are unique to the reference or instance. In this task, at first, the participant has to choose two of four words provided, that he feels match each other. This subtest was completed only by children 9 years or older, instead of the Word Classes subtest. 

For instance, the participant is given the words “fence”, “window”, “glass”, “carpet” and is asked to choose the two that better fit each other. Then, participants are asked, for instance, “how the words window and glass are related”?

Non-Word (NWR) task [115]. NWR task is considered an important clinical marker of DLD [27,41], and it was used in this study to detect potential language deficits of participants. The NWR is consisted of 32 non-words. The nonwords are divided into four categories of two, three, four, and five syllable pairs each. Non-words were selected from a list by a group of 58 undergraduate psychology students who were asked to judge the wordlikeness of non-words on a scale ranging from 1 (very unlikely to be rated as a real Greek word) to 5 (very likely to be rated as real Greek word) [115]. Instead of single nonwords, pairs were adopted to avoid ceiling effect, especially in older children. Instructions and test stimuli were read out loud by the experimenter at a normal rate. The children were instructed to listen carefully to the stimuli and repeat the pairs of nonwords as accurately as they could. Repetition responses were scored either as correct for each nonword pair (score 1) or incorrect (score 0). The maximum correct score that could be achieved was 16. The Cronbach’s αs of the scale ranged from α = 0.76 to α = 0.81, for different grades. An example of Nonwords of three syllables in Greek is “Βλυχηθμός- Ισκάτης”, and an example of four syllables nonwords is “Λαθηκιδής- Μελίκρατο”. 

Children’s Communication Checklist-2 [108]. Is a 70-item questionnaire completed by a caregiver or school teacher and is suitable to detect communication difficulties in children from 4 to 16 years old. The caregiver/school teacher rates how frequently several types of behavior are observed. The response scale is treated as a 4-point Likert scale; less than once a week (or never). At least once a week, but not every day (or occasionally), Once or twice a day (or frequently), and Several times (more than twice) a day (or always). 

It is a very useful tool for screening for language impairment, pragmatic impairment and is an indicator for further investigation of autism disorder. It consists of 10 different subscales (speech, syntax, semantics, coherence, inappropriate initiation, scripted language, use of context, nonverbal communication, social relations, and interests). The test manual reports internal consistency and inter-rater agreement for all scales. Internal consistency was 0.65 or more for all scales. 

The test was not available in the Greek language and for this reason, it was translated back and forth from English to Greek by two native language speakers of each language before used. Checklist translated version agrees with the translation given from a thesis titled “The Adaptation of Children’s Communication Checklist [108] written in Greek language and implemented in parents of 4–7 years old children” [116]. 

The two composite scores of CCC-2 were used, namely General Communication Composite (GCC) and Social-Interaction Deviance Composite (SIDC). GCC is designed to discriminate between children with communication impairments and typically developing children and SIDC is derived to give optimal discrimination between children with typical DLD and those with pragmatic difficulties that are disproportionate to their structural language abilities. Usually, children in the autism spectrum score high in GCC (55 or below), while they present deficits in the SIDC (scores 8 or below). Children with DLD also typically present impairments on GCC (score of 55 or below), however, they score higher on the SIDC (9 or above) [117,118]. This proposed categorical division of symptoms was decided that fits this study’s hypothesis and selected for use.

### 2.3. Statistical Analysis

#### 2.3.1. Preliminary Analysis

Before comparing data between groups, univariate normality was tested for the four groups’ performance on each task and scale. Participants’ performances were normally distributed. Specifically, the histogram of standardized residuals indicated that the data contained approximately normally distributed errors, as did the normal Q–Q plot of standardized residuals, which showed points that were generally on the line. By visually checked the Q–Q plots for the model, the assumption of normality of the residuals is met. However, there were some minus individual outliers detected but they produced only small deviations of the normality. For this reason, outliers are not excluded from the analysis. Multivariate normality was also checked and heteroscedasticity was acceptable in all cases. 

Furthermore, Cronbach’s alpha was calculated to be informed about the internal consistency of the scales used. The CCC-2 subscale consisted of 70 items, α = 0.884, which indicates a high level of internal consistency of the scale with this specific sample. 

We also tested to see if the data meet the assumption of collinearity indicated that multicollinearity was not a concern as the VIF’s of the scales was under 10 (Field, 2013). IQ-Total, VIF = 1.54; CCC-2 (speech) VIF = 4.58; CCC-2 (syntax) VIF = 5.3; CCC-2 (semantic) VIF = 3.84; CCC-2 (coherence) VIF = 4.76; CCC-2 (Inappropriate initiation) VIF = 4.61; CCC-2 (stereotyped) VIF = 4.87; CCC-2 (use of context) VIF = 4.60; CCC-2 (social) VIF = 2.62; CCC-2 (nonverbal) VIF = 2.63; CCC-2 (interests) VIF = 3.99); Multicollinearity was not also a concern for Words-Non Words Total (VIF = 8.5).

Next, multiple comparisons were contacted to detect highly correlated measures and items. It appeared that some factors of the CCC-2 were highly correlated and therefore it was decided that the two basic subcategories of CCC-2 will be used [117]. Specifically, the General Communication Composite (GCC; VIF = 1.05) of CCC-2, designed to discriminate between children with communication impairments and typically developing children and also the Social-Interaction Deviance Composite (SIDC; VIF = 1.05), derived to give optimal discrimination between children with typical DLD and those with pragmatic difficulties that are disproportionate to their structural language abilities, were used. This proposed categorical division of symptoms was decided that fits this study’s hypothesis and selected for use. A full correlation matrix among measures is provided (Table 1).

#### 2.3.2. Power Analysis

An a priori power analysis was conducted using G*Power3 [119] to test the difference between three independent group means using analysis of covariance, a large effect size (d = 0.40), and an alpha of 0.05. The result showed that a total sample of approximately *n* = 110 is acceptable for the study, to achieve a power of 0.80. Our total sample consists of 103 children, who participated in this study.

#### 2.3.3. Main Analysis

Furthermore, to study whether the relation between our participants is explained by group differences, or if there are differences within the groups of participants, we decided to proceed to a linear mixed model (LMM) analysis [120]. LMM technique provides the researcher with the opportunity to simultaneously study both within person (intra-individual) systematic change (level 1) and also between-person (inter-individual) differences (level 2) of the participants in different measures [121]. 

Undoubtedly, participants’ performance differs significantly and heterogeneity is observed among their responses on several tasks. Via this technique we can examine the total between- and within-person variance in the dependent variables. We adopted this analytic strategy to provide answers to the amount of outcome variation located in intra- and inter-individual levels. The intra-class correlation coefficient (ICC) provides the opportunity to examine to what extent the total outcome variation is related to inter-individual differences. Technically speaking, ICC refers to the proportion of the total variation of the dependent variable that can be attributed to between-person differences. The ratio of the between-cluster variance to the total variance is what is called the Intraclass Correlation and provides information of the proportion of the total variation that is accounted by the clustering.

A high ICC (>0.10) implies that the between-group variability dominates the within-group variance, meaning that, most of the differences that we observe across individuals on a variable are stemming from group differences [122]. On the contrary, a low ICC indicates that the variation seen in a variable is the result of individual differences within groups.

Participants IQ total score and age (in months) were treated as covariates, as one-way ANOVA revealed significant group differences of the two variables. Specifically, there appears to be a statistically significant effect of group on the dependent variables of IQ total variable, F (3,99) = 6.3, *p* < 0.001 (between groups DLD and TD children, and ASD-LI and TD children. Age variable was significantly differed as well [F (3,99) = 11.21, *p* < 0.001]. ASD group differed from ASD-LI group, DLD group form the group ASD-LI and ASD-LI from TD children’s group. The means and standard deviation scores are presented in Table 2. Sex was not treated as a covariate as there was no effect of sex or interaction between group and sex. In addition, the educational level of the parents (mothers, fathers) of the participants was also examined and did not produced significant differences between groups, *p* = 0.962 and *p* = 0.298, respectively. Similarly, the socioeconomic status of the family, was also not significantly different among groups, *p* = 0.975. Descriptive statistics of participants’ characteristics are presented in Table 3.

Our concern was that any group of differences may not be due to the group factor, but instead may simply reflect differences in total IQ score and/or age. To address this concern, we performed two LMM analyses for each of the dependent variables (language measures and the composites scores of the CCC-2). In all models A we included measures of total IQ and age as covariates, so that any fixed effect of group then reflects differences due to group membership above and beyond any individual differences in IQ score or age. In all models B we included only the factor of the group. Additionally, in all analyses we considered subjects as random effects. All models A showed that total IQ score and age are not significant predictors of the slope. In other words, the effect of factor group on the dependent variable was constant across the values of total IQ and age. 

## 3. Results

In this study, the measures used to answer the research questions were the EREL, the Non-Words Repetition task (NWR), and also the Children’s Communication Checklist- Second edition (CCC-2). The Non-Words Repetition task (NWR) data was treated as raw scores because we wanted to include in our methodological design the factor of age. However, we were not able to manipulate all data in this way, since there are measures that provide their diagnostic algorithms, and transformation to z scores is already constructed by the manual provided, as in the case of WASI, and CCC-2. For these instruments, the manual’s diagnostic instructions were followed.

The administration of EREL language assessment revealed a language-impaired subgroup of children within the ASD group. The ASD group was then sub-divided upon participants’ performance on the EREL instrument. A group of children with language impairment (ASD-LI), and a group of children with normal language (ALN) were identified; ASD participants whose scores were −1.25 SD or more below the normative mean on two out of four Core Language scores [20] formed the ASD-LI group. The means and standard deviations of groups’ performance on EREL are provided in Table 2. 

These results transformed the experimental groups and an additional group emerged by the analysis. Especially, the final groups were formed as follows: 16 children consisted the ASD group (without additional language impairment) (16 males, 0 females), 28 children the DLD group (17 males, 11 females), 24 children consisted the ASD-LI group (22 males, 2 females), and 35 children (24 males, 11 females) the TD group. Four children from the general population were also found to meet the DLD diagnostic criteria. The group descriptions that emerged by the language ability analysis are presented in Table 2.

**Figure 3:**
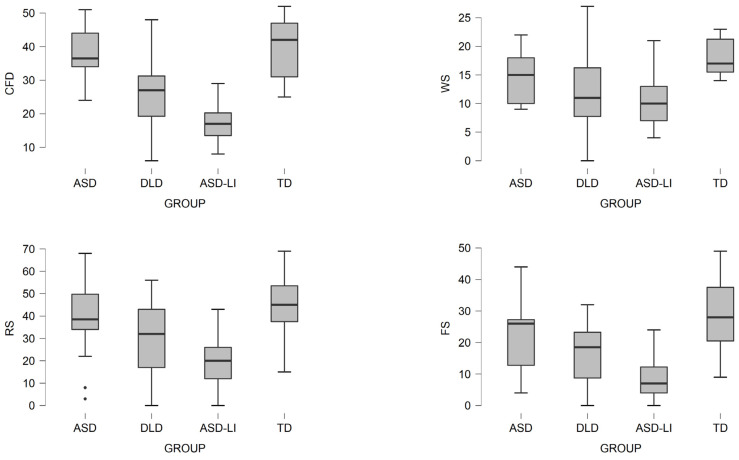

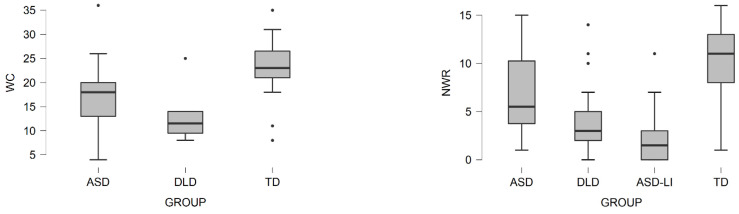
Boxplots for all language measures. CFD = Concept and following directions; WS = Word structure; RS = Recalling sentences; FS = Formulated sentences, WC = Word classes; NWR = Non-word repetition task.

In addition, the multilevel model was preferred for the analysis, as we assumed the existence of heterogeneity between individuals. The results are presented in Table 4. In addition, boxplots upon all the language measures, support this assumption (see Figure 3). Specifically, boxplots indicate important differences between the different groups and, in addition, provide a significant picture of important individuals differences in each group. The results are further supported by the model ICC.

The model ICC was also significant for all the language measures (see Table 3), as the ICC percentages ranged between 23% and 57% on different language tasks, supporting that an important amount of the total variance, can be explained by inter-individual differences. Important differences have been noted between groups at all language measures as well.

For the Concept and Following Directions (CFD) subscale of EREL, results revealed important group differences, *p* < 0.001. Specifically, the ALN group scored significantly higher (*p* < 0.01) from ASD-LI, *p* < 0.001, and DLD children, *p* < 0.001. Interestingly, there were not any important differences between ALN and TD groups of children (*p* = 0.33). No important differences were also observed between participants in the ASD-LI and DLD groups *p* = 0.90, while ASD-LI and also DLD groups, scored significantly lower than the TD groups, *p* < 0.001. The ICC model for CFD measure was large (57%), further supporting the existence of important inter-individual differences among the participants. 

Similarly, important group differences were found for the Word Structure (WS) subscale of EREL, *p* < 0.001. Important differences were found between the ASD-LI group, which scored significantly lower (*p* < 0.01) than the TD group, and also the DLD group scored significantly lower than the TD group, *p* < 0.001. No important differences were found among ALN and TD groups (*p* = 0.07), DLD group (*p* = 0.89), nor ASD-LI group (*p* = 1.0). Similarly, no important differences were observed between ASD-LI and DLD participants (*p* = 0.78). In addition, the ICC model for the WS measure was high (24%), further supporting the existence of important inter-individual differences among the participants. 

As it concerns the Recalling Sentences (RS or SRT) subscale, important group differences were also found, *p* < 0.001. As in the case of the WS subscale, important differences were located in the groups of ASD-LI group which scored significantly lower (*p* < 0.001) than the TD group, and also DLD group scored significantly lower than the TD group, *p* = 0.02. No important differences were found among ALN and TD groups (*p* = 0.50), DLD group (*p* = 0.66), nor ASD-LI group (*p* = 0.07). Similarly, no important differences were observed between ASD-LI and DLD participants (*p* = 0.29). Even not all significantly important, differences revealed for all groups, boxplots presented, indicate for different performances between groups. 

This finding is moreover sustained by the ICC model, which declares a high percentage for RS measure (34%) and outbids the existence of important inter-individual differences in groups. Formulating Sentences (FS) EREL measure has also provided important group differences on language domain, *p* < 0.001. Specifically, important differences were found between the ASD-LI group which scored significantly lower (*p* < 0.01) than the TD group, and also the DLD group (M = 16.61, SD = 8.74) scored significantly lower than the TD group, *p* < 0.001. Important differences were also found between the ALN and TD groups, *p* < 0.01., DLD group (*p* = 0.66), nor ASD-LI group (*p* = 0.07). However, no important differences were observed between ASD-LI and DLD participants (*p* = 0.99), ALN and DLD groups (*p* = 0.97), nor ASD-LI and ALN participants (*p* = 0.98). The ICC model for the subscale of FS was large enough (37%), further supporting the existence of important inter-individual differences among the participants.

The last subscale of EREL measure, Word Classes (WC) also showed important differences between groups, *p* < 0.01. Important differences came up between DLD children, which scored significantly lower (*p* < 0.01) than TD children. No important differences emerged between ALN and DLD, *p* = 0.34, or TD group, *p* = 0.19. Importantly, not an ASD-LI group was formed in this subscale of EREL. WC is a subscale administered only in children between the ages of 9 and 12 years old. None of our participants were found to belong in the ASD-LI group in this age range. Our sample consists of 36 children (from 103) belonging to that age range, of which 19 children are TD participants. The variability of participant’s responses in each group is demonstrated, and it is further supported by a high ICC model for this variable (23%) and indicates important inter-individual differences.

Important differences between groups on language domain are also found on the NWR task, *p* < 0.001. Specifically, differences are observed in ASD-LI children (M = 2.16, SD = 2.73), who scored significantly worse than TD children (M = 10.2, SD = 4.71), *p* < 0.001. Likewise, DLD children (M = 3.79, SD = 3.45), also performed worse than TD children on the same task, *p* < 0.001, and controversially, no significantly different from the ASD-LI group, *p* = 0.99. No differences were also found in the ALN group (M = 6.88, SD = 4.54), related to the ASD-LI (*p* = 0.53), DLD (*p* = 0.30), and TD (*p* = 0.05) group, respectively. The ICC model for NWR is also high (36%), informing once again for inter-individual differences among participants.

**Figure 4:**
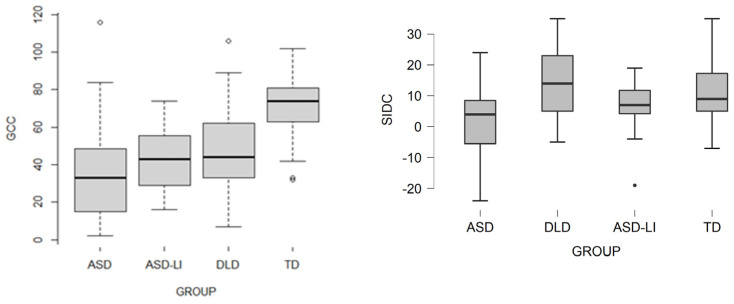
Boxplots for Children’s Communication Checklist-2 composites. GCC = General Communication Composite Children’s Communication Checklist-2; SIDC = Social-Interaction Deviance Composite Children’s Communication Checklist-2.

As it concerns the two composite scores of CCC-2 important findings emerged. GCC subscale (see Figure 4) significantly differentiated between groups, *p* < 0.001. ALN group (M = 37.5, SD = 30.64), the score was significantly lower than TD group (M = 72.06, SD = 17.60), *p* < 0.001, indicating increased deficits in the communication domain. In addition, the ASD-LI group (M = 41.91, SD = 15.74), also obtained significantly elevated communication deficits in comparison to the TD group of children, *p* < 0.001. Finally, important differences were found for DLD children (M = 48.41, SD = 21.8), who demonstrate more impairments in communication in comparison to TD children, *p* < 0.001. No important differences are observed among participants on ASD-LI and DLD groups (*p* = 0.16), who scored similarly on this subscale. No important differences are also found between ALN and ASD-LI (*p* = 0.87), or ALN and DLD (*p* = 0.67) participants.

SIDC subscale of CCC-2 (see Figure 4), also produces important differences among groups, *p* < 0.01. ALL children (M = 1.47, SD = 11.58) differ significantly from DLD (M = 13.44, SD = 12.06), *p* < 0.01, and also TD (M = 9.97, SD = 9.61) children, *p* = 0.03. No important group differences were found between ASD-LI (M = 6.73, SD = 7.96) and ALN, DLD, or TD groups. DLD group also is not importantly different from the TD group (*p* = 0.75).

Related to the two composites of CCC-2, the model ICC was significant for the variable GCC (27%), indicating that the General Communication Composite contributes to the group differences, while the SIDC’s contribution is also important, even if not that large (11%). In sum, the ICC model showed that there are important between-group differences, explain a significant proportion of the observed variance (Table 3).

## 4. Discussion

The results from this study are important and thought-provoking. We had investigated a potential relationship between ASD and DLD, and our findings are indicative of its existence. Specifically, we had expected that an ASD-LI subgroup would have derived from the general ASD group, indicating for language impairments within the ASD population, resembling a language profile similar to DLD children. We had also hypothesized that ASD (ASD-LI and ALN) and DLD groups of children would have scored lower than the TD group on language measures, and also that ASD-LI and DLD groups would demonstrate poorer performance than ALN and TD groups on EREL measures. In addition, we have predicted that the ALN group would have scored poorer than the TD group. We also hypothesized that both the ASD-LI and DLD groups would have performed significantly worse on NWR and SRT tasks.

Most of these hypotheses have been met. Specifically, as it concerns language tasks, a common profile of ASD and DLD is supported by our results, indicating the presence of a language phenotype in ASD similar to that of DLD. Additionally, ASD-LI and DLD groups scored worse than ALN and TD groups on language measures. However, despite our projections, ALN and TD groups revealed no important differences in language tasks. Controversially, ASD-LI and DLD groups produced significantly more deficits than TD groups. Concerning NWR and SRT tasks, our findings on both tasks showed that ASD-LI so as in the DLD group, produced more deficits than the TD group. Nonetheless, despite our expectations, there were not found any important differences between ALN and ASD-LI or DLD groups, or between ALN and TD groups.

Our results are strongly supported by previous studies. Specifically, there is a common profile of ASD and DLD, which indicates a possible common feature between the two developmental disorders, namely, ASD-LI. These findings are in line with previous studies, reporting that a statistically important number of children with ASD, score similarly to DLD children on different language tasks [15], and also that an ASD-LI group exists within the ASD population, which has found to be more impaired on language tasks than ALN children [14,68]. Our results further support the presence of a language phenotype in ASD similar to that of DLD, that is, presenting numerous and serious dysfunctions in several aspects of language [15,68,123].

Moreover, not that surprisingly, ALN and TD groups revealed no important differences on language tasks, providing further support at the assumption of the existence of a subtype inside the ASD population, referred to as the “ASD-LI individuals”. This finding is further supported by De Fossé and colleagues’ [124] study, who found similar asymmetry of frontal language cortex between the ALN and TD control group, indicating that the Broca’s area asymmetry reversal, is stronger connected with language impairment than autism itself.

In the majority of language tasks administered, the ASD-LI group scored lower than the DLD group. This finding could be explained by the fact that ASD-LI children except for the language impairment presented, have additional deficits to manage (e.g., social deficits). This complexity of symptoms with additional impairments in different fields constitutes an additional obstacle in their effort for success on different tasks. 

A special reference is worth making on the RS (SRT) subscale of the EREL instrument. The group differences showed that ASD-LI as well as the DLD group produced more deficits than the TD group. The finding that the clinical groups with LI perform worse than the typical groups is in line with other studies (e.g., [27,32]). Surprisingly, though, there were not found to be any important differences between the ALN and ASD-LI or DLD groups, or between the ALN and TD groups. The means and standard deviations of the task show differences between the groups, whereas the ALN group scores were higher than both the language-impaired groups, though not statistically important. However, our results are in line with Conti-Ramsden et al.’s [27] statements, who argue that sentence repetition could be the strongest clinical marker for the identification of DLD, and can significantly differentiate language-impaired groups from typical language ability, as did in our study. A repetition task considers contributing to the identification of children’s weaknesses and strengths in the language domain [37]. Indeed, in our study SRT managed to reveal differences in both language-impaired groups in comparison to the TD group.

Similarly, the NWR task produced important differences among participants in different groups. Both the language-impaired groups demonstrated worse performance than the TD group. The results of the present study are consistent with several previous studies, supporting that children with ASD-LI and those with DLD have similarly poor performance on the NWR task. Evidence supporting NWR deficits in ASD-LI is compelling and the results of this study confirmed previous findings in the area [13,15,18,125].

Furthermore, the ICC model for all language tasks showed that there are important between-group differences, explaining a significant proportion of the observed variance. This finding provides additional support to the assumption that DLD and ASD constitute points on a continuum of the same disorder instead of separate conditions [19]. Indeed, the experimental groups present great inter-participants’ heterogeneity, as the ICC model shows for all language tasks, suggesting that they do not constitute separate solid groups with distinct traits and symptomatology, but rather are highlighting substantial overlap amongst groups with “distinct” diagnoses [117].

Especially, our results support the existence of an intermediate case of shared symptoms, namely ASD-LI, which indicates an important connection between ASD and DLD. Interestingly, a great number of our participants fell in this intermediate category, not only about the language domain. This finding significantly supports the assumption of existing comorbidity between ASD and DLD. Important relations have been also found to exist between the clinical groups, as revealed by the majority of the tasks and measures employed. These relations, further support an overlapping situation between ASD and DLD. For instance, ASD-LI and DLD groups were found to have similar performance in all tasks of the language domain. This finding points to a comorbid relation in linguistics. Our results allow us to argue in favor of ASD and DLD as disorders lying on a continuum of symptoms, supporting the second theory presented in Figure 2, that the two disorders consist of different manifestations of the same pathology, and lie on the same continuum.

**Figure 5:**
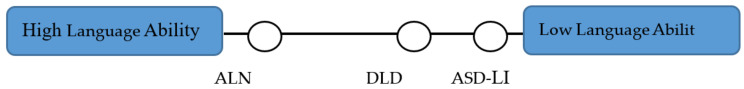
An iconic representation of language ability of children with ASD and DLD.

As illustrated in Figure 5, the group of children with ASD with high language ability (ALN) resides at the one end of the line while, DLD and ASD-LI groups are located in proximity at the other end of the line of the continuum (low language ability). It is important to mention, that the points on the continuum are not related to our numeric data. The clinical groups are only visually illustrated in this figure to facilitate the overall understanding of this theory. We further expected DLD children to present difficulties primarily on their general communication ability as it is tested by the CCC-2, while ASD children would have demonstrated additional impairments in the social interaction subscale (SIDC) of CCC-2 [108], based on the primary deficits of each disorder. Both of our hypotheses have been met.

The CCC-2 instrument identifies children with disproportionate pragmatic and social impairments. In this study this assumption is confirmed, as the ASD group of children were more deficit in this composite, proposing increased pragmatic impairments, unrelated to one’s structural language ability. It is of great importance the finding of different impairments characterized by the fundamental deficits presented by each group, based on CCC-2 composites. The DLD group presented deficits on the General Communication Composite (GCC), and fewer deficits on the SIDC subscale confirming DLD’s children diagnosis, and also the ASD group has demonstrated impairments in GCC and SIDC simultaneously, as it is expected for individuals in the autistic spectrum. The results on GCC show that no important differences are observed in the three clinical groups related to their GCC symptoms. This result was expected, as both developmental conditions are proposed to score similarly on GCC (scores below 55), significantly lower than TD groups [117,118]. The findings for SIDC were partially expected. Specifically, the ALN group of children presented more social deviance deficits, which was excepted and our hypothesis was met. Still, no other experimental group did, either ASD-LI group, which was assumed to present significantly important deficits in the social deviance domain, as ALN did.

Nevertheless, our results are confirmed from previous studies supporting that the SIDC score is expected to be eight or less in children with ASD [117,118]. Importantly, the ASD-LI group also scored below eight on the SIDC composite. At the other end of the distribution, children with DLD usually score above eight, as in our study [117,118]. Our findings gain support from a validation study [117], in which the GCC was effective in distinguishing between children with communication impairments (including both DLD and autism) and typically developing children. The SIDC was usually less than eight in children with ASD.

Nevertheless, these findings raise questions about the extent to which pragmatic impairments are a secondary consequence of speech and language disorder. For instance, a large proportion of cognitively able children with ASD experience additional language impairments [15,126]. On the other hand, non-autistic children with LI are not only characterized by structural language deficits, but many also experience difficulties with pragmatic aspects of language [117]. In this study DLD group of children was found free of social- pragmatics deficits as it was captured by the SIDC composite of CCC-2. 

It is apparent that language impairment adversely affects social interaction. However, the present data show that some children have pragmatic impairments out of keeping with their structural language skills. This is in line with previous studies showing that children with good structural language can nevertheless have pragmatic difficulties [127]. At a theoretical level, to further investigate these language—and consequently—communicative abilities is critical as communication deficits have been related to difficulties in understanding other’s minds and in social functioning [128,129,130].

### 4.1. Limitations

It is, finally, important to acknowledge some methodological limitations. Evidence supporting NWR deficits in ASD-LI are numerous and the results of this study are consistent with several previous studies in the area [13,15,18,125]. In more recent studies that have compared individuals with ASD-LI and those with DLD on NWR tasks, results provided evidence for significant differences in the error patterns between these two groups. We did not directly compare the error patterns in the ASD-LI and DLD groups of children. In addition, we did not measure articulation or oro-motor skills in the ASD and DLD children. Therefore, the NWR deficits could be attributed to oro-motor deficits in the ASD-LI or DLD children, rather than explained by cognitive deficits. 

Nevertheless, a study comparing NWR performance in ASD and DLD [125] included only DLD children with no referral of articulation impairments and still found poor NWR in these children. Therefore, impairments related to the NWR in DLD may not solely be explained by concurrent articulation deficits. Nevertheless, future research should continue to attempt to illustrate the specific cognitive and linguistic relation to poor NWR and SNR in ASD-LI and DLD children, to further inform the debate on etiological overlap in these two developmental conditions. 

Bias can be introduced at any phase of a research process. We tried to prevent systematic errors and bias during designing, conducting, and reporting the results of our study. However, we believe some selection bias was inserted in the study due to the fact that participants, especially children in pediatric groups, were recruited through a call from speech pathologists and special educators who knew children. 

### 4.2. Strengths of the Study

This is the first study, to our knowledge, conducted in Cyprus investigating the ASD and DLD population directly. The prevalence of ASD and DLD individuals in Cyprus is still not determined even if Special Education Services needs are elevated. This study succeeded to locate and examine an important number of ASD and DLD individuals all over the country, given the small numbers of the population in Cyprus. This study could be considered as attaining numbers of participants similar to what we could find in an epidemiological study in Cyprus, by addressing these two developmental conditions in a wide range of childhood ages. 

In addition, our results are also significant, as they confirm the existence of this intermediate set of symptoms that consists of the ASD-LI group, in a different language such as the Greek language. Greek, is a language with significant peculiarities and complexity in expression. The view that this middle ground exists is further reinforced by our data. This category of mixed symptoms deserves to be investigated closely, to obtain optimal results, both in research and clinical practice. The assumption that this intermediate group does, indeed, exist will no longer cause stress to specialists working with clinical groups about what is the most appropriate intervention plan to follow. Instead, they will be provided with the necessary support and guidance to work in the light of this middle cluster of symptoms.

To further investigate the existence and relation of the two disorders will provide important and valuable information scientifically and clinically. The need for the development of effective intervention programs for both developmental conditions is highlighted by the specialists of all fields working with these populations, as the number of the affected children increases daily.

## 5. Conclusions

In conclusion, our results further support the assumption of common symptomatology of the two disorders and are in line with the suggestion of moving beyond the “labels” and start subgrouping individuals in response to their characteristics, instead of trying to detect the symptoms following an already given diagnosis. The apparent need to divide the ASD group into two different categories based on the divergence of symptoms presented, namely ASD children with additional language impairment and ASD children without, is an indicative starting point of the need to change our perspectives and methodologies. Beyond that, if we accept that this category exists, it is important to provide answers to consequent questions. For instance, at what point in the continuum can we place this “shared” category of social symptoms? Can anyone argue that it is placed exactly in the middle of the two predominant disorders? Is it possibly closer to autism disorder, or perhaps to language disorder? With all this in mind, unquestionably, the research community has still a lot to discover and many challenges to overcome concerning this particular category.

## Figures and Tables

**Table 1:** Correlations among measures.

Variable	CFD	WS	RS	FS	WC	NWR	SIDC	GCC
CFD	—							
WS	0.632 **	—						
RS	0.712 **	0.698 **	—					
FS	0.806 **	0.741 **	0.891 **	—				
WC	0.328	—	0.184	0.425	—			
NWR	0.679 **	0.674 **	0.662 **	0.694 **	0.393	—		
SIDC	−0.101	0.026	−0.149	−0.055	−0.195	−0.177	—	
GCC	0.172	0.208	0.342 **	0.123	0.139	0.206	−0.324	—

** Significant at *p* = 0.001. Note: CFD = Concept and Following Directions; WS = Word Structure; RS = Recalling Sentences; FS = Formulated Sentences; WC = Word Classes EREL measures; NWR = Non-Word Repetition Task; SIDC = Social-Interaction Deviance Composite, Children’s Communication Checklist-2; GCC = General Communication Composite, Children’s Communication Checklist-2.

**Table 2:** Descriptive statistics of group formation.

	ASD (*n* = 16)			ASD-LI (*n* = 24)			DLD (*n* = 28)			TD (*n* = 35)		
	Mean	SD	Range	Mean	SD	Range	Mean	SD	Range	Mean	SD	Range
Age	108.81	23.33	73–140	78.29	6.85	72–99	97.21	19.98	72–193	105.71	23.79	72–144
IQ Total	92.38	10.56	85–126	86.88	5.36	80–103	90.18	9.54	71–111	96.43	8.8	76–113
CFD	37.69	7.41	24–51	17.04	5.44	8–29	24.79	9.89	6–48	40.37	8.33	25–52
WS	14.57	5.16	9–22	10.46	4.25	4–21	12.2	6.09	0–27	18.06	3.4	14–23
FS	22.31	11.66	4–44	9	6.49	0–24	16.61	8.74	0–32	29.51	10.71	9–49
RS	39.13	17.47	3–68	18.33	10.31	0–43	30.82	16.08	0–56	45.89	12.43	15–69
WC	18.33	9.06	4–36	-	-		12.75	5.47	8–25	23.16	6.32	8–35

Note: CFD = Concept and Following Directions; WS = Word Structure; RS = Recalling Sentences; FS = Formulated Sentences; WC = Word Classes.

**Table 3:** Participants’ Characteristics.

	ASD (*n* = 16)			ASD-LI (*n* = 24, girls = 1)			DLD (*n* = 28, girls = 10)			TD (*n* = 35, girls = 11)		
	Mean	SD	Range	Mean	SD	Range	Mean	SD	Range	Mean	SD	Range
Age *	108.81	23.33	73–140	78.29	6.85	72–99	97.21	19.98	72–193	105.71	23.79	72–144
IQ-Total	92.38	10.56	85–126	86.88	5.36	80–103	90.18	9.54	71–111	96.43	8.8	76–113
SES	1.56	0.51	1–2	1.58	0.50	1–2	1.61	0.50	1–2	1.60	0.50	1–2
EducM	1.75	0.58	1–3	1.79	0.78	1–3	1.68	0.72	1–3	1.71	0.72	1–3
EducF	1.63	0.72	1–3	1.80	0.88	1–4	1.67	0.68	1–3	1.44	0.56	1–3

Note: SES = Socio-Economic Status, EducM = Mothers’ Educational Level, EducF = Fathers’ Educational Level. * Age is presented in months.

**Table 4:** Intra-class correlation and confidence intervals (CI) for Language Measures and CCC-2 Composites.

Measure	ICC	CI
Language Domain		
NWR	0.36	1.827–9.694
CFD	0.57	17.883–42.053
WS	0.24	10.053–17.516
RS	0.34	20.438–46.656
FS	0.37	9.746–28.988
WC	0.23	11.253–25.089
GCC	0.27	33.032–67.143
SIDC	0.11	2.585–13.449

Note: ICC = Intra-Class Correlation Coefficient, CI = 95% Confidence Interval. NWR = Non-Word Repetition task; CFD = Concept and Following Directions; WS = Word Structure; RS = Recalling Sentences; FS = Formulated Sentences, WC = Word Classes; GCC = General Communication Composite Children’s Communication Checklist-2; SIDC = Social-Interaction Deviance Composite Children’s Communication Checklist-2.

## Data Availability

The data presented in this study are available on request from the corresponding author, GS. The data are not publicly available due to their containing information that could compromise the privacy of research participants.
